# Prognostic impact of diffuse large B-cell lymphoma with extra copies of MYC, BCL2 and/or BCL6: comparison with double/triple hit lymphoma and double expressor lymphoma

**DOI:** 10.1186/s13000-019-0856-7

**Published:** 2019-07-17

**Authors:** Sixia Huang, Lin Nong, Wei Wang, Li Liang, Yalin Zheng, Jumei Liu, Dong Li, Xin Li, Bo Zhang, Ting Li

**Affiliations:** 1 Department of Pathology, Peking University First Hospital, 8 Xishiku Street, Xicheng District, Beijing, 100034 China; 2 Department of Pathology, Peking University Health Science Center, Beijing, 100191 China

**Keywords:** Diffuse large B-cell lymphoma, Extra copies, MYC, BCL2, BCL6

## Abstract

**Background:**

The poor outcome of high-grade B-cell lymphoma, with rearrangements of MYC, BCL2 and/or BCL6, also known as double-hit lymphoma or triple-hit lymphoma (DHL or THL), has been well documented, while the clinical significance of extra copies of MYC, BCL2 or BCL6 are still less well known.

**Methods:**

In total, 130 cases of diffuse large B-cell lymphoma, not otherwise specified (DLBCL-NOS) were included in our study. Fluorescence in situ hybridization and immunohistochemistry were performed in all cases to evaluate the genetic status and protein expression levels of MYC, BCL2 and BCL6.

**Results:**

Among the 130 cases of DLBCL, the prevalence rates of extra copies of MYC, BCL2 and BCL6 were 10.8, 20.0 and 14.6%, respectively, and the corresponding rates of gene rearrangement were 10.0, 14.6 and 16.9%, respectively. In total, 7.7% (10/130) of patients were DHL/THL; 9.2% (12/130) of patients were DLBCL with MYC and BCL2 and/or BCL6 gene abnormalities including rearrangements or extra copies, while excluded DHL/THL. The positive protein expression rates of MYC, BCL2 and BCL6 were 46.9% (61), 75.4% (98) and 70.0% (91), respectively. Among the 51 cases with MYC/BCL2 co-expression, 14 cases showed concurrence of MYC, BCL2 and/or BCL6 genetic abnormalities, and the remaining 37 cases were classified as double-expressor lymphoma (DEL). MYC and BCL2 rearrangement and BCL2 extra copies were all associated with upregulated protein expression. Cases with concurrence of MYC, BCL2 and/or BCL6 genetic abnormalities were both associated with MYC/BCL2 co-expression. Patients with concurrence of MYC, BCL2 and/or BCL6 genetic abnormalities excluded DHL/THL had shorter OS (*P* < 0.001) than patients with DLBCL with no genetic change, and showed no statistical different with patients with DHL/THL (*P* = 0.419). Extra copies of MYC was independent prognostic factors for DLBCL.

**Conclusions:**

Patients with MYC and BCL2 and/or BCL6 gene extra copies might show a trend towards poor prognosis, and the detection of extra copies of MYC, BCL2 and BCL6 might deserve more attention.

**Electronic supplementary material:**

The online version of this article (10.1186/s13000-019-0856-7) contains supplementary material, which is available to authorized users.

## Background

Diffuse large B-cell lymphoma, not otherwise specified (DLBCL-NOS) (hereinafter referred to as the “DLBCL”) is by far the most common type of non-Hodgkin lymphoma worldwide, accounting for 30–40% of all lymphomas. DLBCL consists of a group of highly heterogeneous tumours with different clinicopathological characteristics, genetic alterations, responses to therapy and prognosis. The standard first-line therapy for patients with DLBCL is R-CHOP (rituximab, cyclophosphamide, doxorubicin, vincristine and prednisone) chemotherapy, and approximately 65% of patients achieve a complete response (CR) [1]. Approximately 30–40% of patients still progress to relapsed/refractory disease, and it is important to accurately identify patients at high risk for relapse or lack of response to therapy. Several prognostic factors, such as the cell of origin (COO) and the International Prognostic Index (IPI), have already been identified. In addition, several key cytogenetic alterations and the abnormal expression of certain proteins have also been shown to affect the treatment response and clinical outcome of patients with DLBCL [2–4].

Recently, MYC, BCL2 and/or BCL6 rearrangements and protein expression levels were identified as prognostic factors in DLBCL, especially for MYC [4–6]. As the deregulation of MYC has both gene activating and repressing functions that can lead to many genetic abnormalities, such as mutations in TP53 [7]. The clinical significance of MYC and BCL2 and/or BCL6 rearrangement for patients with DLBCL has been well elucidated; these rearrangements promote highly aggressive clinical behaviour with resistance to standard chemotherapy and extremely poor outcomes [6, 8]. In 2016, the revised World Health Organization (WHO) guidelines for tumours of haematopoietic and lymphoid tissues classified this type of tumour in a new category named high-grade B-cell lymphoma with rearrangements of MYC and BCL2 and/or BCL6, also called “double-hit” (DHL) or “triple-hit” lymphoma (THL) [9]. In total, approximately 7–10% of patients with DLBCL were classified as having DHL/THL. Among them, approximately 65% of patients were classified as having MYC/BCL2 type DHL; 14% had MYC/BCL6 type DHL; and the remaining 21% of patients had THL [10]. In addition, patients with MYC and BCL2 protein co-expression who did not harbour genetic alterations were defined as having “double-expressor” lymphoma (DEL), which accounted for approximately 19–34% of cases of DLBCL [5]. Previous studies showed that DEL was also associated with a poor outcome intermediate between those of DHL and DLBCL after treatment with R-CHOP [11].

In addition to genetic translocations, multiple other mechanisms could also cause increased MYC protein expression in patients with DLBCL, such as copy number alterations. Extra copies of MYC were identified in approximately 2–20% of patients with DLBCL [6]. Green TM et al. [11] found that extra copies of MYC and BCL2 were both prognostic factors for DLBCL, while several studies showed discordant findings [12]. Up to now, the prognostic role of extra copies of MYC, BCL2 or BCL6, as well as the concurrence of MYC, BCL2 and/or BCL6 genetic abnormalities in DLBCL were still unclear. Hence, in our study, fluorescence in situ hybridization (FISH) and immunochemistry (IHC) were performed in 130 cases of DLBCL to evaluate the genetic status and protein expression levels of MYC, BCL2 and BCL6. Incidence and survival analyses were performed to evaluate the effects of extra copies of the three genes, and comparisons with DHL/THL and DEL were also conducted.

## Materials and methods

### Sample selection

All 130 patients with DLBCL-NOS diagnosed between 2014 and 2018 were selected from the Department of Pathology, Peking University First Hospital. All 130 formalin-fixed paraffin-embedded (FFPE) samples were stored at room temperature, and the materials were sufficient and available. All tumours were reclassified according to the 2016 WHO classification system by two experienced haematopathologists and all tumours showed no EBV infection. All cases were further divided into the following five categories: (1) DHL/THL [9], which was defined as DLBCL with MYC and BCL2 and/or BCL6 rearrangements per the classic definition; (2) atypical DHL/THL [13], which included all DLBCLs with concurrent MYC and BCL2 and/or BCL6 gene abnormalities other than typical DHL/THL, with two subgroups, one with MYC rearrangement and BCL2 and/or BCL6 extra copies and one with MYC extra copies and BCL2 and/or BCL6 rearrangement/extra copies; (3) DEL, which was defined as those with MYC and BCL2 protein co-expression but not DHL/THL or atypical DHL/THL; and (4) conventional DLBCL, which was defined as DLBCL with no genetic alterations. The clinical information of all the patients was obtained by reviewing the records in the digital medical database; 92 patients had available clinical data, and 108 patients had complete follow-up data. The selection of therapy for these patients was determined by the physician, and the therapy included R-CHOP, R-CHOPE (R-CHOP and etoposide) and R-EPOCH (rituximab, etoposide, prednisone, vincristine, cyclophosphamide, and doxorubicin). In our study, R-CHOPE and R-EPOCH were classified as intensive chemotherapy.

### Fluorescence in situ hybridization (FISH)

FISH was performed on 130 DLBCL FFPE samples following the manufacturer’s instructions. The probes used were the MYC (8q24) break apart rearrangement probe (F.01054; Anbiping, China); BCL2/IGH fusion translocation t (14; 18) probe (F.01066, Anbiping, China) and BCL6 (3q27) break apart rearrangement probe (F.01069, Anbiping, China). For the MYC and BCL6 genes, samples with two fusion signals were classified as normal; those with three or four fusion signals, with or without separate signals, were determined to be gains; those with five fusion signals or more, with or without separate signals, were classified as amplification; and the appearance of separate signals with one green signal and one red signal was classified as a rearrangement. For the BCL2 gene, samples with two red signals targeting BCL2 and two green signals targeting IGH were classified as normal; those with three or four targeted separate signals, with or without fusion signals, were determined to be gains; those with five or more targeted separate signals, with or without fusion signals, were classified as amplifications; and the presence of fusion signals was classified as a rearrangement. The probe signals for a monolayer of at least 200 tumour cell nuclei were counted per sample at X100 magnification, and genetic alterations were determined when they exceeded a 20% threshold in the number of nuclei [13].

### Immunohistochemistry (IHC)

IHC was performed on a total of 130 samples using the DAKO EnVison detection Kit (Dako, Glostrup, Denmark) in accordance with the manufacturer’s instructions. Freshly cut 4-um FFPE sections were subjected to heat-induced antigen retrieval in EDTA buffer (PH = 9.0) for 2–3 min. A panel of primary antibodies was utilized in our study, including anti-CD10 (clone 56C6, 1:50, Novocastra), anti-BCL6 (clone PG-B6p, 1:40, Dako), anti-MUM1 (clone MUM1p, 1:50, Dako), anti-BCL2 (clone 124, 1:100, Dako), anti-C-MYC (clone EP121, 1:75, Zhongshan) and anti-P53 (DO-7, 1:100, Dako). IHC staining was performed with colour development carried out using 3,3′-diaminobenzidine tetrahydrochloride.

According to the requirements and recommendations of the 2016 revised WHO classification system, the expression statuses of BCL2 and C-MYC were identified in all samples. Cutoff values of 50% for BCL-2 expression and 40% for C-MYC expression was used to identify DEL [9]. Germinal centre B-cell (GCB)/non-GCB types were grouped according to Han’s algorithm [14], which is based on immunostaining against CD10, BCL-6 and MUM1, using a cut-off of 30% for each antibody.

### Statistical analysis

The relationships between genetic alterations and clinicopathological characteristics were analysed by chi-squared tests or Fisher’s exact tests. The clinical outcome was represented by overall survival (OS), which was defined as the period from the day of diagnosis to the day of death or the last follow-up. Estimates of OS were calculated via the Kaplan-Meier method, and the comparison of differences between the OS of the two groups was evaluated by the log-rank test. Univariate and multiple Cox regression models were used to determine the prognostic risk factors of DLBCL. All statistical analyses were performed with SPSS 20.0 software. All *p*-values in this study were 2-sided. In order to increased the statistical significance and forecasted the prognostic factors of DLBCL, a corrected *P*-value < 0.003 (0.05/17) by Bonferroni multiple testing corrections was considered significant in multiple Cox regression models, and other statistical analysis results considered significant with a *p*-value < 0.05.

## Results

### Protein expression levels and the incidence of extra gene copies and gene rearrangements

**Fig. 1:**
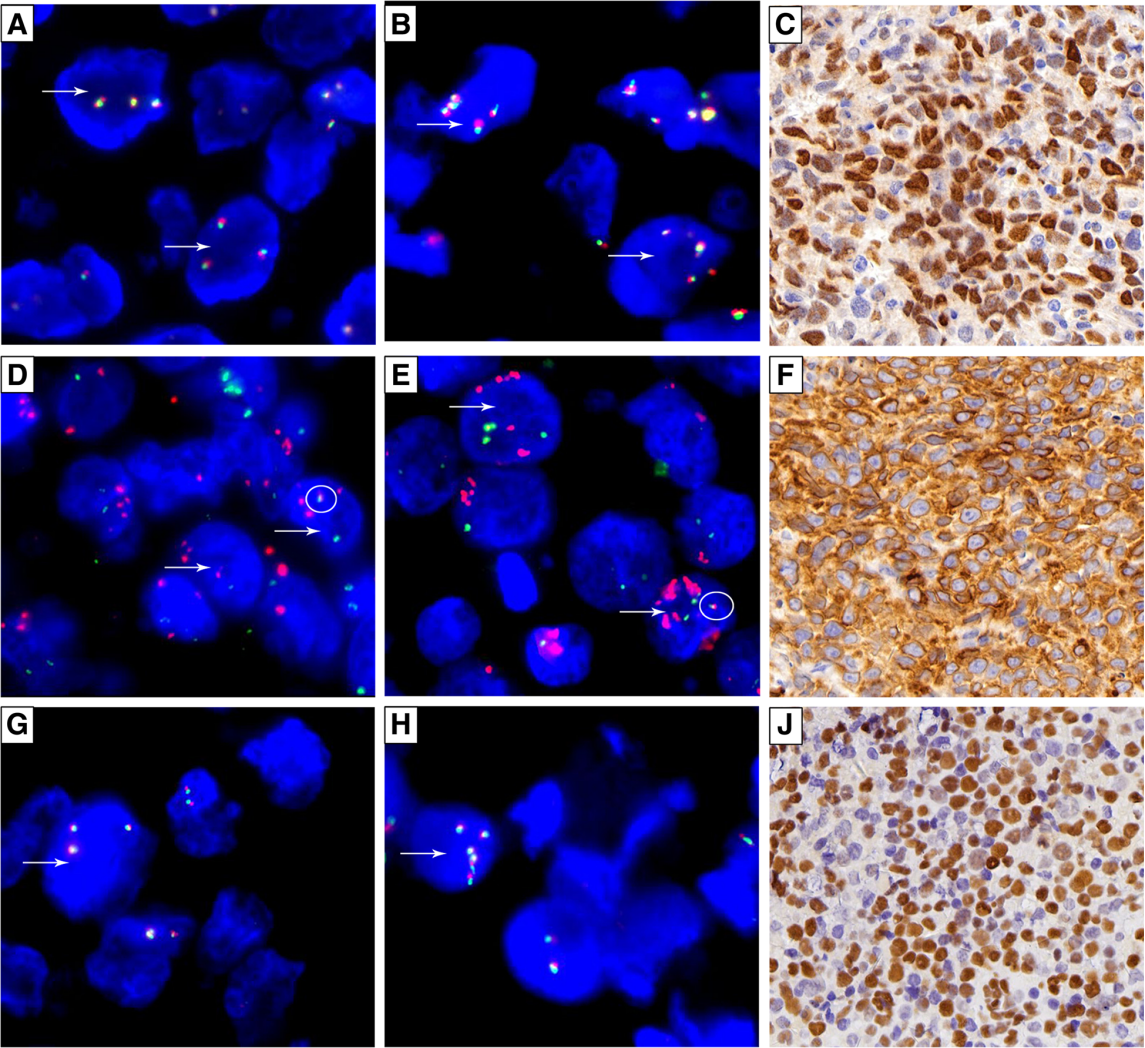
Extra copies and protein expression levels of MYC, BCL2 and BCL6. White arrows indicate the gain of MYC (**a**), BCL2 (**d**), BCL6 (**g**) and amplification of MYC (**b**), BCL2 (**e**) and BCL6 (**h**). White circles represent BCL2 translocation. Patients with both BCL2 extra copies and rearrangement are shown (**d**, **e**) (×1000). Immunohistochemistry showed positive expression of MYC (**c**), BCL2 (**f**) and BCL6 (**i**) (× 400)

The 130 patients comprised 64 males and 66 females, with ages ranging from 2 to 86 years (median 60 years). In total, 50 of the 130 (38.5%) patients were diagnosed with GCB-type DLBCL, whereas 80 of the 130 (61.5%) patients were diagnosed with non-GCB-type DLBCL. All 130 samples subjected to FISH and IHC showed usable results. Extra copies of MYC were identified in 14 (10.8%) samples, including 13 (10.0%) with MYC gain and 1 (0.8%) with MYC amplification (Fig. 1a, b). MYC translocation was shown in 13 (10.0%) samples; and the other 103 (79.2%) samples had normal MYC loci. Extra copies of BCL2 were identified in 26 (20.0%) samples, including 22 (16.9%) with gains and 4 (3.1%) with amplification (Fig. 1d, e); 19 (14.6%) cases samples BCL2/IGH translocations. Among the samples with BCL2 genetic abnormalities, 3 (2.3%) showed the co-existence of extra BCL2 copies and BCL2 rearrangements (Fig. 1d, e). The remaining 88 (67.7%) samples had a normal BCL2 status. Extra copies of BCL6 were identified in 19 (14.6%) samples, including 18 (13.8%) with gains and 1 (0.8%) with amplification (Fig. 1g, h); 22 (16.9%) samples had BCL6 rearrangements. Among the samples with BCL6 genetic abnormalities, 3 (2.3%) showed the co-existence of extra copies and rearrangements of BCL6. In addition, 92 (70.8%) samples had normal BCL6 genes.

DHL was identified in 8 (6.2%) samples, including 4 of MYC/BCL2 type DHL and 4 of MYC/BCL6 type DHL, while THL was identified in 2 (1.5%) samples. Seven (5.4%) patients were classified as having atypical DHL, and 5 (3.8%) patients were classified as having atypical THL. Among the patients with atypical DHL, 3 had extra copies of both MYC and BCL2, 1 had extra copies of both MYC and BCL6, 2 had extra copies of MYC and BCL2 rearrangement, and 1 had extra copies of MYC and the co-existence of extra copies and rearrangements of BCL2. Among the patients with atypical THL, 2 had extra copies of MYC, BCL2 and BCL6; 1 had extra copies of MYC and BCL2 and BCL6 rearrangement; 1 had MYC rearrangement and extra copies of both BCL2 and BCL6; and 1 had extra copies of both MYC and BCL6 and BCL2 extra copies and rearrangements.

At the protein level, the positive rates of the expression of MYC, BCL2 and BCL6 were 46.9% (61/130), 75.4% (98/130) and 70.0% (91/130), respectively (Fig. 1c, f, i), and the positive rate of MYC/BCL2 co-expression was 39.2% (51/130). Among 51 patients with MYC/BCL2 co-expression, 7 had DHL/THL, 7 had atypical DHL/THL, and the remaining 37 had DEL. DEL accounted for 34.3% of all DLBCLs other than DHL/THL and atypical DHL/THL.

### The relationships between extra copies of MYC, BCL2, and BCL6 and clinicopathological features

#### Immunophenotype

Table 1 summarizes the relationships between MYC, BCL2 or BCL6 genetic alterations and protein expression. Patients with DLBCL with MYC rearrangement (84.6%) more frequently showed MYC expression than patients with DLBCL with a normal MYC gene (40.8%) (*P* = 0.006), while patients with DLBCL with extra MYC copies (57.1%) had no difference in MYC expression compared to patients with normal MYC genes (*P* = 0.245). Patients with DLBCL with extra copies (92.3%) and rearrangement (94.7%) of BCL2 both more frequently expressed BCL2 than did patients with DLBCL with normal BCL2 genes (67.0%) (*P* = 0.011, *P* = 0.021). Patients with DLBCL with extra copies (68.4%) and rearrangement (86.4%) of BCL6 both showed no relationship with BCL6 expression compared to patients with DLBCL with normal BCL6 genes (67.4%) (*P* = 0.930, *P* = 0.115). Compared to patients with DLBCL with MYC, BCL2 or BCL6 rearrangements, those with DLBCL with extra copies of the three genes all tended towards lower rates of the expression of the corresponding protein, although no significant differences were found (*P* = 0.209, *P* > 0.999, *P* = 0.255, respectively).

**Table 1:** The relationship between MYC, BCL2 and BCL6 genetic alterations and protein expression

	MYC +	P	BCL2 +	P	BCL6 +	P	MYC/BCL2 +	P	GCB	non-GCB	P
MYC normal	42(40.8)		76(73.8)		73(70.9)		36(35.0)		39(37.9)	64(62.1)	
MYC EC	8(57.1)	0.245	11(78.6)	> 0.999*	10(71.4)	> 0.999*	6(42.9)	0.563	6(42.9)	8(57.1)	0.719
MYC R	11(84.6)	0.006*	11(84.6)	0.513*	8(61.5)	0.528	9(69.2)	0.031*	5(38.5)	8(61.5)	0.967
MYC EC VS R		0.209*		> 0.999*		0.695*		0.252*			0.816
BCL2 normal	36(40.9)		59(67.0)		62(70.5)		26(29.5)		34(38.6)	54(61.4)	
BCL2 EC	16(61.5)	0.064	24(92.3)	0.011*	17(65.4)	0.622	16(61.5)	0.003	7(26.9)	19(73.1)	0.274
BCL2 R	12(63.2)	0.077	18(94.7)	0.021*	15(78.9)	0.579*	12(63.2)	0.005	11(57.9)	8(42.1)	0.123
BCL2 EC VS R		0.987		> 0.999*		0.495*		0.987			0.027
BCL6 normal	40(43.5)		69(75.0)		62(67.4)		33(35.9)		35(38.0)	57(62.0)	
BCL6 EC	12(63.2)	0.118	16(84.2)	0.555*	13(68.4)	0.930	11(57.9)	0.074	7(36.8)	12(63.2)	0.922
BCL6 R	11(50.0)	0.580	16(72.7)	0.826	19(86.4)	0.115*	9(40.9)	0.660	10(45.5)	12(54.5)	0.523
BCL6 EC VS R	11(64.7)	0.296	14(82.4)	0.451*	11(64.7)	0.255*	10(58.8)	0.187	6(35.3)	11(64.7)	0.676
Conventional DLBCL	23(37.1)		42(67.7)		44(71.0)		18(29.0)		26(41.9)	36(58.1)	
Atypical DHL/THL	8(66.7)	0.107*	10(83.3)	0.491*	9(75.0)	> 0.999*	7(58.3)	0.049	6(50.0)	6(50.0)	0.606
DHL/THL	8(80.0)	0.016*	9(90.0)	0.262*	8(80.0)	0.716*	7(70.0)	0.027*	5(50.0)	5(50.0)	0.633
Atypical DHL/THL VS DHL/THL		0.646*		> 0.999*		> 0.999*		0.675*			> 0.999*
DEL +	37(100.0)	< 0.001*	37(100.0)	< 0.001*	25(67.6)	0.878	37(100.0)	< 0.001*	10(27.0)	27(73.0)	0.156
DEL -	8(11.3)		42(59.2)		49(69.0)		0(0)		29(40.8)	42(59.2)	
Atypical DHL/THL VS DEL		0.002*		0.056*		0.731*		< 0.001*			0.140

*EC* extra copies, *R* rearrangement, *DLBCL* diffuse large B-cell lymphoma, *DHL* double hit lymphoma, *THL* triple hit lymphoma, *DEL* double expressor lymphoma, *GCB* germinal center B-cell

^*^Fisher’s exact test

Compared to patients with conventional DLBCL, patients with DHL/THL more often expressed the MYC protein (*P* = 0.016), and patients with DHL/THL and atypical DHL/THL both more often co-expressed MYC/BCL2 (*P* = 0.049, *P* = 0.027). Patients with DHL/THL had a higher MYC/BCL2 co-expression rate (70%) than patients with atypical DHL/THL (58.3%), although no significant difference was found (*P* = 0.675). Compared to patients with DEL, patients with atypical DHL/THL less often expressed MYC and co-expressed MYC/BCL2 (*P* = 0.002, *P* <0.001).

Patients with genetic alterations of MYC, BCL2 or BCL6, DHL/THL, atypical DHL/THL or DEL all showed no difference with conventional DLBCL in COO. Compared to BCL2 rearrangement, DLBCL with extra copies of BCL2 more often had a non-GCB type. The positive expression rates of P53 in DHL/THL, atypical DHL/THL and conventional DLBCL were 0% (0/9), 50.0% (5/10) and 17.4% (4/23), respectively.

#### Clinical features

In total, 92 patients had complete and available clinical information. In total, 52.2% (48/92) of patients accepted R-CHOP/R-CHOP-like therapy; 33.7% (31/92) of patients accepted intensive chemotherapy, 10.9% (10/92) of patients only underwent resection, and 3.3% (3/92) of patients did not accept any therapy. Among the atypical DHL/THL patients, the rate of CR in those treated with intensive chemotherapy was 75.0% (3/4), which was higher than the rate (33.3%, 2/6) in patients treated with R-CHOP; however, the difference was not statistically significant (*P* = 0.524). Among the patients with DHL/THL, the rate of CR (50%, 1/2) in patients treated with intensive chemotherapy was also higher than the rate (0%, 0/5) in patients treated with R-CHOP (*P* = 0.103).

The relationships between extra copies of genes and clinical features, as well as the clinical differences between atypical DHL/THL and DHL/THL or DEL, are shown in Table 2. Extra copies of MYC were more frequently found in patients with B symptoms (*P* = 0.020). Patients with extra copies of BCL2 had more marrow involvement (*P* = 0.002). Extra copies of BCL6 were more often identified in male patients (*P* = 0.047). Patients with atypical DHL/THL more frequently had marrow involvement (*P* = 0.004). There were no clinical differences between patients with DHL/THL and atypical DHL/THL or DEL.

**Table 2:** The relationship between MYC, BCL2 and BCL6 genetic alteration and clinical features

	MYC EC			BCL2 EC			BCL6 EC			Atypical DHL/THL			Atypical DHL/THL	DHL + THL	P	Atypical DHL/THL	DEL	P
	+	–	P	+	–	P	+	–	P	+	–	P						
	n(%)	n(%)		n(%)	n(%)		n (%)	n(%)		n(%)	n(%)		n(%)	n(%)		n(%)	n(%)	
	*n* = 82			*n* = 81			*n* = 78			*n* = 49			*n* = 18			*n* = 32		
Age																		
Median (range)	60(18–69)	61(2–86)		61(2–80)	60 (18–86)		57 (28–82)	63(2–86)		61(28–69)	63(29–86)		61(28–69)	60 (54–73)		61(28–69)	63(28–86)	
> 60	4(36.4)	37(52.1)	0.519*	11(52.4)	29(48.3)	0.749	7(38.9)	35(58.3)	0.147	5(50.0)	23(59.0)	0.609	5(50.0)	3(37.5)	0.664*	5(50.0)	12(54.5)	0.811
Sex																		
Male	6(54.5)	30(42.3)	0.445	12(57.1)	24(40.0)	0.174	12(66.7)	24(40.0)	0.047	6(60.0)	13(33.3)	0.156*	6(60.0)	5(62.5)	> 0.999*	4(40.0)	9(40.9)	> 0.999*
Female	5(45.5)	41(57.7)		9(42.9)	36(60.0)		6(33.3)	36(60.0)		4(40.0)	26(66.7)		4(40.0)	3(37.5)		6(60.0)	13(59.1)	
Ann Arbor stage> 2	10(90.9)	51(71.8)	0.274*	17(81.0)	43(71.7)	0.565*	14(77.8)	43(71.7)	0.766*	9(90.0)	26(66.7)	0.244*	9(90.0)	8(100.0)	> 0.999*	9(90.0)	16(72.7)	0.387*
Extranodal sites≥2	6(54.5)	38(53.5)	0.949	14(66.7)	32(53.3)	0.288	11(61.1)	31(51.7)	0.481	5(50.0)	19(48.7)	0.942	5(50.0)	6(75.0)	0.367*	5(50.0)	12(54.5)	0.811
Elevated serum LDH	7(63.6)	33(47.8)	0.518*	9(45.0)	32(54.2)	0.475	9(52.9)	26(44.1)	0.518	6(60.0)	19(48.7)	0.725*	6(60.0)	5(62.5)	> 0.999*	6(60.0)	14(66.7)	> 0.999*
ECOG PS ≥ 2	2(18.2)	13(18.3)	> 0.999*	5(23.8)	13(21.7)	0.839	4(22.2)	11(18.3)	0.739*	2(20.0)	7(17.9)	> 0.999*	2(20.0)	5(62.5)	0.145*	2(20.0)	3(13.6)	0.637*
IPI ≥ 3	7(63.6)	35(49.3)	0.520*	11(52.4)	33(55.0)	0.836	9(50.0)	31(51.7)	0.901	6(60.0)	18(46.2)	0.496*	6(60.0)	7(87.5)	0.314*	6(60.0)	13(59.1)	> 0.999*
B symptoms	9(81.8)	29(40.8)	0.020*	13(61.9)	26(43.3)	0.143	9(50.0)	28(46.7)	0.804	7(70.0)	16(41.0)	0.157*	7(70.0)	6(75.0)	> 0.999*	7(70.0)	10(45.5)	0.265*
Marrow involvement	3(27.3)	6(8.5)	0.097*	7(33.3)	3(5.0)	0.002*	4(22.2)	9(15.0)	0.483*	4(40.0)	1(2.6)	0.004*	4(40.0)	6(75.0)	0.188*	4(40.0)	4(18.2)	0.218*
Nervous involvement	2(18.2)	7(9.9)	0.347*	5(23.8)	5(8.3)	0.064	2(11.1)	4(6.7)	0.617*	2(20.0)	2(5.1)	0.180*	2(20.0)	1(12.5)	> 0.999*	2(20.0)	2(9.1)	0.572*
Treatment																		
R-CHOP	7(63.6)	35(49.3)		12(57.1)	29(48.3)		8(44.4)	33(55.0)		6(60.0)	21(53.8)	0.444	6(60.0)	5(62.5)	0.460	6(60.0)	9(40.9)	0.662
R-CHOPE	4(36.4)	24(33.8)		8(38.1)	19(31.7)		7(38.9)	19(31.7)		4(40.0)	10(2.6)		4(40.0)	2(25.0)		4(40.0)	11(50.0)	
Resection	0(0)	10(14.1)		1(4.8)	9(15.0)		2(11.1)	7(11.7)		0(0)	7(17.9)		0(0)	0(0)		0(0)	1(4.5)	
No therapy	0(0)	2(2.8)		0(0)	3(5.0)		1(5.6)	1(1.7)		0(0)	1(2.6)		0(0)	1(12.5)		0(0)	1(4.5)	
ASCT (total)	2(18.2)	12(16.9)	> 0.999*	3(14.3)	10(16.7)	> 0.999*	2(11.1)	9(15.0)	> 0.999*	1(10.0)	4(10.3)	> 0.999*	1(10.0)	2(25.0)	0.559*	1(10.0)	6(27.3)	0.387*
Treatment response																		
CR	5(45.5)	37(55.2)	0.132	9(42.9)	32(57.1)	0.458	9(56.3)	30(51.7)	0.786	5(50.0)	21(56.8)	0.071	5(50.0)	1(12.5)	0.207	5(50.0)	10(47.6)	0.096
PR	3(27.3)	25(37.3)		8(38.1)	18(32.1)		5(31.3)	23(39.7)		2(20.0)	14(37.8)		4(50.0)	2(20.0)		2(20.0)	10(47.6)	
SD	3(27.3)	5(7.5)		4(19.0)	6(10.7)		2(12.5)	5(8.6)		3(30.0)	2(5.4)		3(30.0)	3(37.5)		3(30.0)	1(4.8)	
Relapse	3(27.3)	10(14.7)	0.377*	4(19.0)	11(19.3)	> 0.999*	4(23.5)	9(15.5)	0.475*	3(30.0)	6(16.2)	0.377*	3(30.0)	3(37.5)	> 0.999*	3(30.0)	2(9.5)	0.296*

*EC* extra copies, *DHL* double hit lymphoma, *THL* triple hit lymphoma, *DEL* double expressor lymphoma, *LDH* lactate dehydrogenase, *ECOG* Eastern Cooperative Oncology Group, *PS* performance status, *IPI* international prognostic index, *ASCT* autologous stem cell transplantation, *CR* complete response/remission, *PR* partial response/remission, *SD*,stable disease

^*^Fisher’s exact test

### Survival analysis

#### Prognostic role of extra copies of MYC, BCL2 or BCL6 and comparison with gene rearrangement

**Fig. 2:**
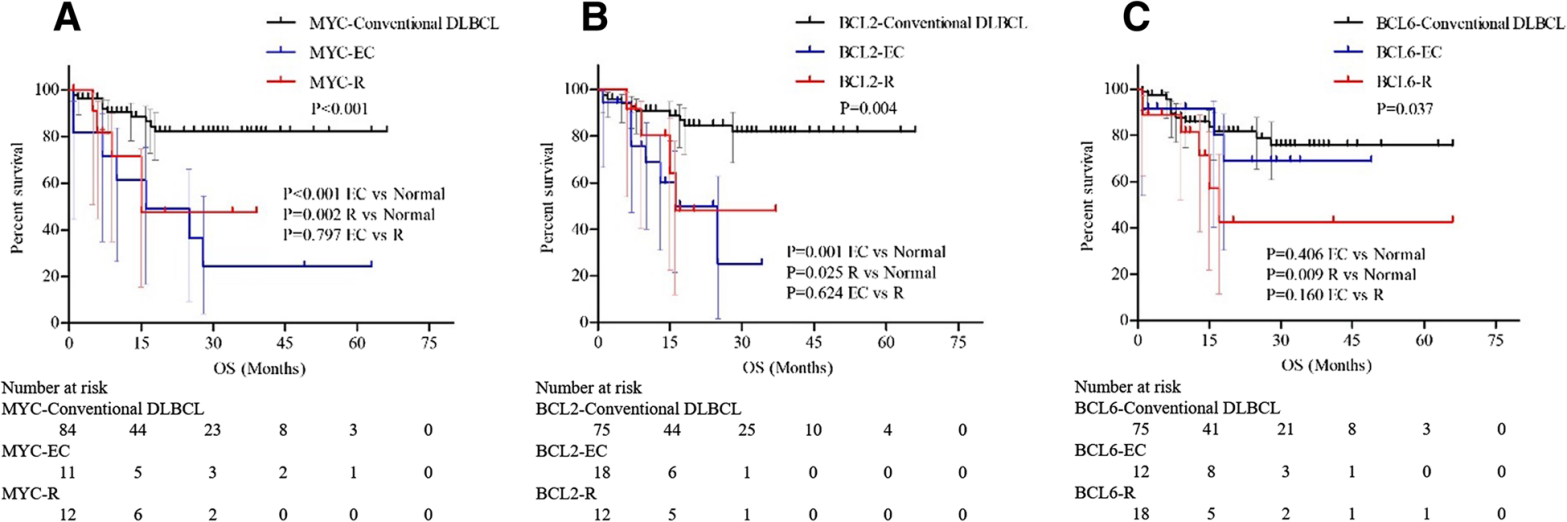
Overall survival comparison between patients with MYC, BCL2 or BCL6 extra copies (EC) and rearrangements (R). (**a**) patients with MYC EC vs patients with MYC R; (**b**) patients with BCL2 EC vs patients with BCL2 R; (**c**) patients with BCL6 EC vs patients with BCL6 R

In total, 108 patients had available follow-up information, and the median follow-up duration was 19.6 months, ranging from 1 to 66 months. Overall, 22.2% (24/108) of patients died due to DLBCL progression, and 3 patients died for other reasons. A total of 19.6% of patients experienced recurrence. The survival analysis showed that patients with MYC extra copies and rearrangement both had shorter OS than patients with normal MYC genes (*P* < 0.001, *P* = 0.002, Fig. 2a). Patients with BCL2 extra copies and rearrangement both had shorter OS than patients with normal BCL2 genes (*P* = 0.001, *P* = 0.025, Fig. 2b). Patients with BCL6 rearrangement had shorter OS than patients with normal BCL2 (*P* = 0.009, Fig. 2c), while there was no difference in OS between patients with BCL6 extra copies and patients with normal BCL6 genes (*P* = 0.406, Fig. 2c).

Compared with the OS of patients with gene rearrangements, the OS of patients with extra MYC copies, extra BCL2 copies, and extra BCL6 copies all showed no significant differences (*P* = 0.797, *P* = 0.624, *P* = 0.160, respectively, Fig. 2a- c).

#### Prognostic role of atypical DHL/THL and comparison with DHL/THL or DEL

**Fig. 3:**
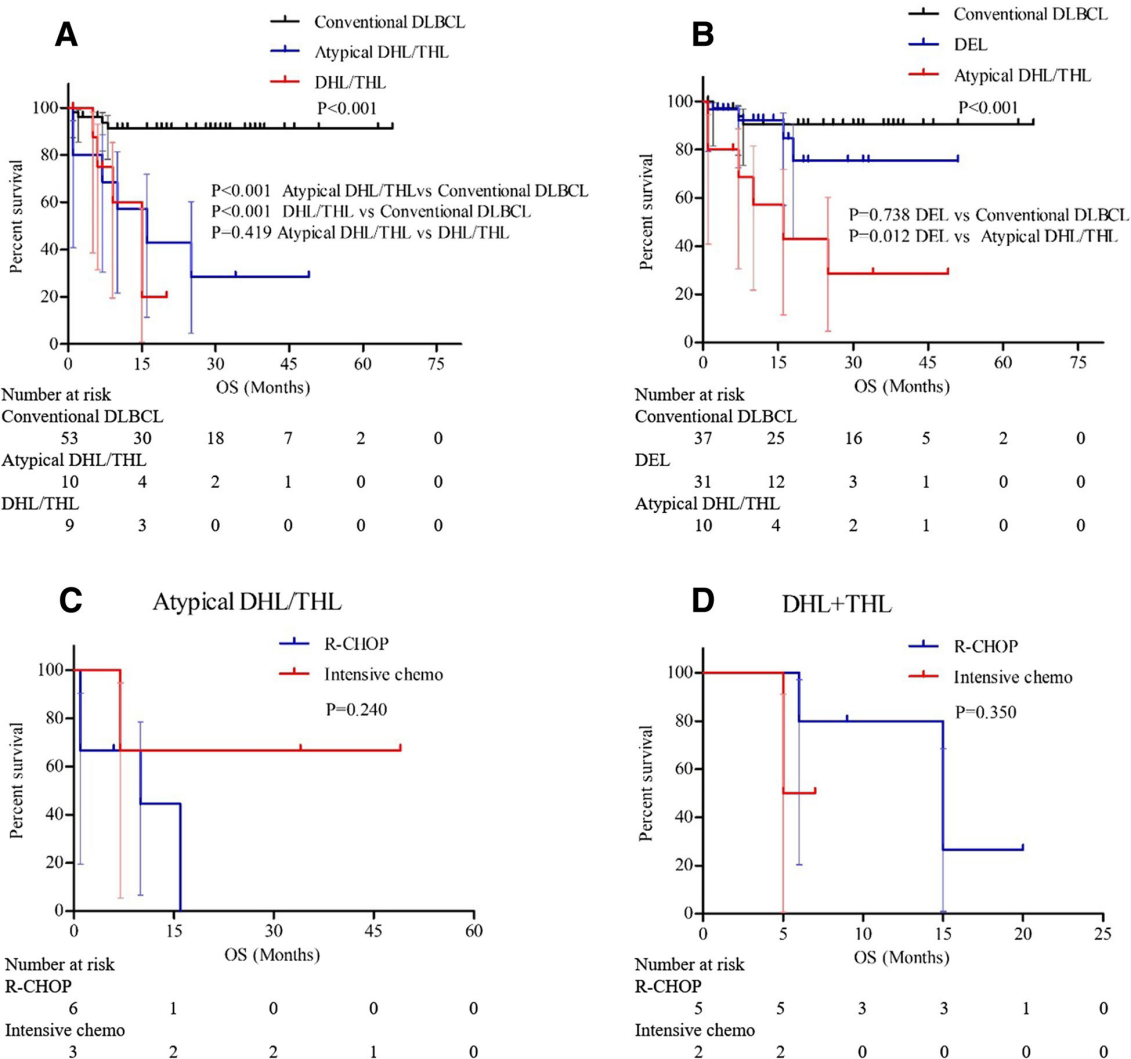
Overall survival comparison between patients with atypical double-hit lymphoma (DHL)/ triple-hit lymphoma (THL) and DHL/THL or double expressor lymphoma (DEL). (**a**) patients with atypical DHL/THL vs patients with DHL/THL; (**b**) patients with atypical DHL/THL vs patients with DEL; (**c**) different induction therapy in patients with atypical DHL/THL; (**d**) different induction therapy in patients with DHL/THL

Five out of seven patients with atypical DHL and 5/5 patients with atypical THL had available follow-up information. Sixty percent (3/5) of patients with atypical DHL died, with a median OS of 17.5 months, and 60% (3/5) of patients with atypical THL died, with a median OS of 16 months. The Kaplan-Meier analysis showed that patients with atypical DHL/THL and DHL/THL both had shorter OS times than patients with conventional DLBCL (*P* < 0.001, P < 0.001, Fig. 3a). And the OS of patients with atypical DHL/THL was not different from that of patients with DHL/THL (*P* = 0.419, Fig. 3a). Besides, patients with MYC/BCL2 type DHL, MYC/BCL6 type DHL and MYC/BCL2 type atypical DHL all had shorter OS than patients with conventional DLBCL (*P* = 0.007, *P* < 0.001, and *P* < 0.001, respectively, Additional file 1: Figure S1).

Patients with DEL showed no statistical difference in OS compared to patients with conventional DLBCL (*P* = 0.738, Fig. 3b). While patients with atypical DHL/THL had a shorter OS than patients with DEL (*P* = 0.012, Fig. 3b). Regarding treatment selection, both for patients with atypical DHL/THL and patients with DHL/THL, no difference was found in OS between those receiving R-CHOP and intensive chemotherapy (*P* = 0.240 and *P* = 0.350, Fig. 3c, d).

#### Multivariate analysis

The variables used in Cox regression models were age, R-CHOP vs R-CHOPE & R-EPOCH, high IPI, bone marrow involvement, high Arbor stage, more than two extranodal sites involved, elevated LDH, MYC, BCL2 or BCL6 genetic changes (rearrangement or extra copies) and protein expression, DHL/THL, atypical DHL/THL and DEL. The results of univariate analysis showed that extra copies of MYC, MYC rearrangement, extra copies of BCL2, BCL6 rearrangement, atypical DHL/THL, and DHL/THL all had prognostic significance (*P* = 0.003, *P* = 0.015, *P* = 0.037, *P* = 0.039, *P* = 0.001 and *P* = 0.004, respectively; Table 3). The multivariate analysis indicated that only extra copies of MYC was independent prognostic factors for DLBCL (*P* = 0.002, Table 3).

**Table 3:** Prognostic factors of OS of patients with DLBCL

	Univariate analysis		Multivariate analysis1	
	P	HR (95%CI)	P	HR (95%CI)
*MYC* EC	0.003	4.694 (1.697–12.988)	0.002	8.068 (2.145–30.348)
*MYC* R	0.015	3.831 (1.302–11.277)	0.028	8.005 (1.248–51.359)
MYC expression	0.290	1.590 (0.674–3.752)	0.956	1.035 (0.307–3.491)
*BCL2* EC	0.037	2.819 (1.063–7.472)	0.172	2.901 (0.629–13.373)
*BCL2* R	0.180	2.224 (0.691–7.155)	0.106	0.089 (0.005–1.676)
BCL2 expression	0.390	1.614 (0.542–4.806)	0.624	0.696 (0.163–2.966)
*BCL6* EC	0.517	1.461 (0.465–4.592)	0.434	1.683 (0.457–6.195)
*BCL6* R	0.039	2.724 (1.049–7.069)	0.550	1.454 (0.426–4.962)
BCL6 expression	0.585	0.768 (0.297–1.981)	0.813	1.174 (0.310–4.446)
Atypical DHL/THL	0.001	9.054 (2.549–32.167)	0.385	2.841 (0.269–29.957)
DHL/THL	0.004	7.011 (1.866–26.342)	0.463	3.111 (0.150–64.521)
DEL	0.740	1.228 (0.366–4.124)	0.825	0.809 (0.123–5.304)
Age	0.860	0.930 (0.417–2.077)	0.442	2.167 (0.301–15.592)
R-CHOP vs R-CHOPE & R-EPOCH	0.731	0.840 (0.310–2.273)	0.080	0.266 (0.061–1.169)
IPI ≥ 3	0.021	3.618 (1.215–10.770)	0.323	0.223 (0.011–4.367)
Marrow involvement	0.007	3.463 (1.415–8.475)	0.011	4.163 (1.436–12.453)
Ann Arbor stage > 2	0.044	7.926 (1.061–59.198)	0.279	3.704(0.345–39.721)
Extranodal sites ≥2	0.044	2.807(1.027–7.678)	0.599	0.603(0.091–3.977)
Elevated LDH	0.050	2.610(0.999–6.819)	0.260	2.470(0.512–11.920)

*DLBCL* Diffuse large B-cell lymphoma, *EC* extra copies, *R* rearrangement, *DHL* double hit lymphoma, *THL* triple hit lymphoma, *DEL* double expressor lymphoma, *IPI* international prognostic index, *LDH* lactate dehydrogenase

## Discussion

DHL/THL is a new type of large B-cell lymphoma that has a poor prognosis, and the importance of detecting MYC, BCL2 and BCL6 rearrangements and protein expression levels in routine clinical work has been acknowledged. However, little is known about the prognostic value of extra copies of these three genes, which might also be noteworthy in DLBCL. In this study, we performed cytogenetic analysis on patients with DLBCL and tried to investigate the prognostic significance of extra copies of these three genes; and we found that patients with atypical DHL/THL might show the potential for a poor outcome.

MYC rearrangement has been proven to be an indicator of poor prognosis in DLBCL in many studies [13, 15]. In patients with DHL/THL, the cooperation between BCL2-induced inhibition of apoptosis and MYC-induced genomic instability promotes oncogenesis and tumour progression. Similar to the results of previous studies, in our study, patients with rearrangements of MYC, BCL2 and BCL6 all showed shorter OS than those with normal genes (Fig. 2a- c). Several earlier studies also analysed the importance of extra copies of MYC and BCL2 in patients with DLBCL and showed conflicting results [12, 13, 16–18]. Quesada et al. [13] studied 663 patients with de novo DLBCL and found that 12% of patients had extra MYC copies, 16% of patients had extra BCL2 copies, and 13% of patients had extra BCL6 copies. However, only patients with extra copies of MYC had a shorter OS. In the study by Lu et al. [16], extra copies of MYC were found in 18 (7.5%) patients, and extra copies of BCL2 were found in 65 (27.1%) patients. Both extra copies of MYC and BCL2 were associated with a shorter OS. While other studies showed conflicting results, they found that extra copies of MYC had no relationship with a poor outcome [12]. In our study, extra copies of MYC, BCL2 and BCL6 were found in 14 (10.8%), 26 (20.0%) and 19 (14.6%) patients, respectively. Similar to the results of previous studies, our results showed that extra MYC copies and extra BCL2 copies were both associated with a short OS (Fig. 2a, b). Extra BCL6 copies had no prognostic significance. We further compared patients with extra gene copies with those with rearrangements, and no difference in OS was found (Fig. 2a- c). Multivariable analyses also showed that extra copies of MYC was independent prognostic factors in patients with DLBCL, indicating the significant prognostic value of extra copies of MYC and BCL2 and suggesting that the detection of gene extra copies should be considered in patients with DLBCL.

Previous studies also explored the definition and prognostic value of atypical DHL/THL [13, 16, 19]. Quesada et al. [13] defined double/triple extra copies lymphoma (DECL/TECL) as tumours with extra copies of MYC and BCL2 and/or BCL6 and tumours with extra MYC copies and concomitant rearrangements of BCL2 and/or BCL6. They found that patients with DECL/TECL showed a similar poor clinical outcome as that of patients with DHL/THL. Li, S et al. [19] defined atypical DHL as all tumours with concurrent MYC and BCL2 abnormalities other than coexisting translocations, and patients with atypical DHL had similar clinical outcomes to patients with DHL. In our study, we expanded the definition of atypical DHL/THL to include all patients with concurrent MYC and BCL2 and/or BCL6 abnormalities except DHL/THL. Our study showed similar results to those of previous studies. Patients with atypical DHL/THL predicted poor outcome (Fig. 3a). Furthermore, we compared atypical DHL/THL with DHL/THL, and we found that atypical DHL/THL showed similar prognostic value to DHL/THL, which was similar to the results of earlier studies [13, 16]. Patients with DEL had a poor prognosis, while in our study, patients with DEL had a similar OS to that of patients with conventional DLBCL (Fig. 3b). This might be because patients with atypical DHL/THL were removed from DEL, and atypical DHL/THL was obviously more aggressive than DEL (Fig. 3b).

Although the results of comparisons of different groups of patients indicated that atypical DHL/THL associated with inferior prognosis, while as the incidences of the extra copies of MYC, BCL2 and BCL6 were low, only 12 patients with atypical DHL/THL were included in our study, making our study exploratory rather than definitive, which suggested that patients with extra copies of MYC and BCL2 and/or BCL6 might showed a trend towards poor clinical outcome. Besides, half of patients with atypical DHL/THL showed P53 overexpression, while none of patients with DHL/THL showed P53 overexpression. And previous study showed that TP53 mutation was proved to be associated with MYC genetic abnormality and P53 overexpression was a predictor for poor clinical outcome [7, 20], so P53 overexpression might also contribute to the inferior prognosis of atypical DHL/THL in our study. While the number of DHL/THL and atypical DHL/THL was limited, so larger cohorts were needed in future studies to verify the above results.

A series of studies have tried to explore the relationship between MYC and/or BCL2 rearrangement and protein expression; patients with DLBCL with MYC or BCL2 rearrangement frequently express the corresponding proteins, and patients with DHL/THL often co-express MYC/BCL2 [13, 21–23]. The frequency of rotein expression was higher than that of cytogenetic changes, and several patients with MYC and/or BCL2 rearrangement showed no protein expression. Our study showed similar results; patients with DLBCL with MYC or BCL2 rearrangements more often showed MYC or BCL2 protein expression. We also found that patients with DLBCL with extra copies of BCL2 more often expressed the BCL2 protein, and patients with atypical DHL/THL and DHL/THL co-expressed MYC/BCL2. Our results indicate that like gene rearrangement, extra copies of MYC and/or BCL2 could also lead to the upregulation of protein expression, while the frequency of protein expression in patients with DLBCL with extra copies of MYC and/or BCL2 was lower than that in patients with DLBCL with gene rearrangement, which was similar to the results of previous studies [13, 21].

Several large retrospective studies have clearly shown that the treatment response to R-CHOP in patients with DHL/THL is suboptimal [24, 25]. Hence, intensified induction chemotherapy was explored as a means of improving the outcome of DHL/THL. To date, the addition of other intensive induction regimens to R-CHOP has been generally accepted as the intensive therapies available, including R-EPOCH and R-Hyper-CVAD. Compared with patients treated with R-CHOP, patients with DHL/THL treated with R-EPOCH had superior OS and event-free survival times [25, 26]. Quesada et al. [13] evaluated the function of intensive chemotherapy in patients with DECL/TECL, and the results showed that patients with DECL/TECL could also benefit from intensive chemotherapy. In our study, R-CHOPE and R-EPOCH were classified as intensive chemotherapy. We found no significant differences between R-CHOP and intensive chemotherapy in patients with DHL/THL or atypical DHL/THL (Fig. 3c, d). This might be because the number of patients with genetic alterations in our study was limited.

In summary, MYC and BCL2 rearrangements and extra BCL2 copies were associated with increased corresponding protein expression levels. DHL/THL and DLBCL with MYC and BCL2 and/or BCL6 gene abnormalities while excluded DHL/THL (atypical DHL/THL) were both associated with MYC/BCL2 co-expression. Extra copies and rearrangements of MYC and BCL2 predicted poor clinical outcomes. Furthermore, extra copies of MYC were independent prognostic factors for DLBCL. Atypical DHL/THL as defined in this study were found in 12 patients, our results suggested that patients with MYC and BCL2 and/or BCL6 gene extra copies might show a trend towards poor prognosis, which require more attention in diagnosis and treatment.

## Additional file

Additional file 1: **Figure S1.** Overall survival comparison between patients with different type of DHL (Double hit lymphoma) or atypical DHL, which defined as DLBCL with MYC and BCL2 or BCL6 gene abnormalities, while excluded DHL. (A) Patients with MYC/BCL2 type DHL vs patients with MYC/BCL6 type DHL; (B) Patients with MYC/BCL2 type atypical DHL vs patients with MYC/BCL6 type atypical DHL. (PDF 2355 kb)

## Data Availability

The data and materials used in the current study are available from the corresponding author on reasonable request.

## Abbreviations

COO: Cell of origin
CR: Complete response
DEL: Double-expressor lymphoma
DHL: Double-hit lymphoma
DLBCL-NOS: Diffuse large B-cell lymphoma, not otherwise specified
FFPE: Formalin-fixed paraffin-embedded
FISH: Fluorescence in situ hybridization
GCB: Germinal centre B-cell
IHC: Immunochemistry
IPI: International Prognostic Index
OS: Overall survival
R-CHOP: Rituximab, cyclophosphamide, doxorubicin, vincristine and prednisone
R-CHOPE: Rituximab, cyclophosphamide, doxorubicin, vincristine, prednisone etoposide
R-EPOCH: Rituximab, etoposide, prednisone, vincristine, cyclophosphamide, and doxorubicin
THL: Triple-hit lymphoma
WHO: World Health Organization
