# Supplementary material for: Prognostic impact of diffuse large B-cell lymphoma with extra copies of MYC, BCL2 and/or BCL6: comparison with double/triple hit lymphoma and double expressor lymphoma
Source: Diagn Pathol. 2019 Jul 17;14:81. doi: 10.1186/s13000-019-0856-7 (PMC6637540; doi:10.1186/s13000-019-0856-7)
Supplement: Supplementary file 1 — Figure S1. Overall survival comparison between patients with different type of DHL (Double hit lymphoma) or atypical DHL, which defined as DLBCL with MYC and BCL2 or BCL6 gene abnormalities, while excluded DHL. (A) Patients with MYC/BCL2 type DHL vs patients with MYC/BCL6 type DHL; (B) Patients with MYC/BCL2 type atypical DHL vs patients with MYC/BCL6 type atypical DHL. (PDF 2355 kb) [file 13000_2019_856_MOESM1_ESM.pdf]

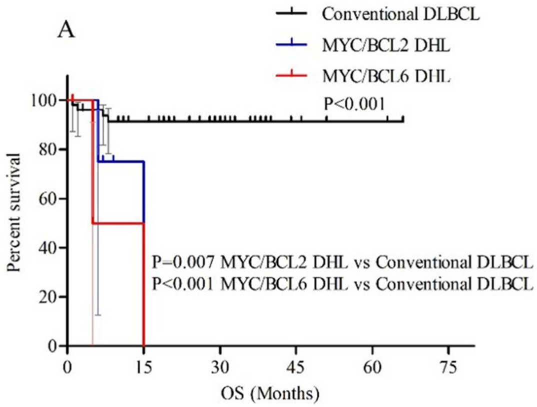

Number at risk

Conventional DLBCL	53	30	18	7	2	0
MYC/BCL2 DHL	4	0	0	0	0	0
MYC/BCL6 DHL	4	0	0	0	0	0

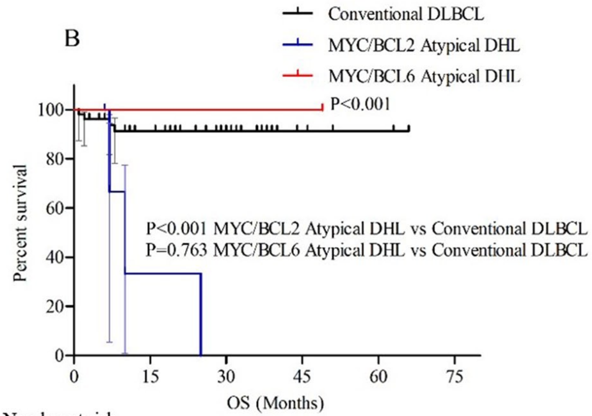

Number at risk

Conventional DLBCL	37	25	16	5	2	0
MYC/BCL2 Atypical DHL	4	1	0	0	0	0
MYC/BCL6 Atypical DHL	1	1	1	1	1	1
